# Understanding the socio-ecological determinants of COVID-19 vaccine uptake: A cross-sectional study of post-COVID-19 Tanzania

**DOI:** 10.4102/jphia.v16i3.1145

**Published:** 2025-04-18

**Authors:** Chima E. Onuekwe, Ambrose T. Kessy, Egidius Kamanyi, Paul E. Kazyoba, Alexander Makulilo, Thomas Ndaluka, Magolanga Shagembe, Asha Hayeshi, Violet Mathenge, Tumaini Haonga, William Mwengee, Grace E. Saguti, Charles Sagoe-Moses

**Affiliations:** 1 Department of Immunizations, Emergency Preparedness and Reponse (EPR), World Health Organisation, Dar es Salaam, United Republic of Tanzania; 2 Centre for Health and Allied Legal and Demographical Development, Research and Training (CHALADDRAT), Nnamdi Azikiwe University, Awka, Nigeria; 3 Directorate of Research, Publications and Consultancy, University of Dodoma, Dodoma, United Republic of Tanzania; 4 Department of Planning, Finance and Administration, The Law School of Tanzania (LST), Dar es Salaam, United Republic of Tanzania; 5 Department of Sociology and Anthropology, Faculty of Social Sciences, University of Dar es Salaam, Dar es Salaam, United Republic of Tanzania; 6 Department of Research and Development, Mabibo Traditional Medicine Research Centre, National Institute for Medical Research, Dar es Salaam, United Republic of Tanzania; 7 Department of Political Science and Public Administration, Faculty of Social Sciences, University of Dar Es Salaam, Dar es Salaam, United Republic of Tanzania; 8 Tanzanian Psychological Association (TAPA), Dar es Salaam, United Republic of Tanzania; 9 Department of Sociology and Anthropology, Dodoma University, Dodoma, United Republic of Tanzania; 10 Department of Immunizations, Emergency Preparedness and Response (EPR), World Health Organization, Dodoma, United Republic of Tanzania; 11 Health Promotion Unit, Ministry of Health, Dodoma, United Republic of Tanzania; 12 Emergency Preparedness and Response (EPR), World Health Organization, Dar es Salaam, United Republic of Tanzania; 13 Department of Leadership and Management, World Health Organization, Dar es Salaam, United Republic of Tanzania

It is no longer any news that COVID-19 swept across the planet, starting with the first confirmed case in Wuhan China in 2019. It is equally no news anymore that COVID-19 has impacted every corner of society, from science and medicine to the economy, politics, religion, race, and culture. The pandemic has proven to be fertile ground not only for widely disparate opinions about the disease but also for information overload. In its wake, the virus overwhelmed hospitals, crippled economies and industries, and forced institutions to change their norms. Globally, more than seven million people died of the disease as of 7 January 2024, including over 257,984 in Africa as of 18 November 2022. Countries, in their bid to respond to the pandemic, introduced restrictions on travel, social gatherings, rapid testing, vaccinations, social distancing, and financial support.^1^

Although COVID-19 has come to stay, the application of various response strategies has widened the knowledge horizon of the disease by more than four years. Different countries have applied different approaches to effectively track and respond to the pandemic and to guide public health efforts, research, and policies. Governments in many countries have implemented significant public health measures to prevent the spread of the virus.^2^ Currently, reliable scientific evidence has unravelled the epidemiology of COVID-19, its transmission, mutations, vaccines, and treatment.

However, one area that remains intriguing and consequential is the social environment of COVID-19 vaccination behaviour. While most countries embraced all measures to stop the outbreak, especially vaccination, Tanzania’s national leadership encouraged alternative traditional dietary remedies and steam inhalation [*kujifukiza*] against globally prescribed vaccines. This position was confirmed further on 2 February 2021, in Dodoma, Tanzania’s capital, when the health minister announced that Tanzania “has no plans to accept COVID-19 vaccines.” A few days later, President John Magufuli expressed doubt about COVID-19 vaccines sourced abroad, insisting that Tanzania would only adopt vaccinations after they were certified by Tanzania’s experts.^3^

As a result, the initial vaccine uptake in Tanzania was comparatively low compared to other neighbouring countries due to perceived low risk and scepticism about the safety of the vaccine. Affirmatively, Konje, Basinda, Kapesa, et al. published in September 2021 that two-thirds of Tanzanian healthcare professionals were hesitant about the COVID-19 vaccine and suggested it could be predictive of low uptake in the general population.^4^

Indeed, it manifested as a low rate of vaccination uptake among Tanzanians. As of January 2022, only 2.8% of the general population had been vaccinated against COVID-19. At the same time, Tanzania was among the top 10 of the 34 countries in Africa for the World Health Organization (WHO), UNICEF, and Gavi concerted Vaccine Delivery Partnership (CoVDP), with less than 10% full vaccination coverage in the general population.

However, a year and a half later, from a poor coverage of 2.8%, Tanzania leapfrogged to 52.5% of the general population, who had completed the primary series of COVID-19 vaccinations. Through this coverage, Tanzania topped the list of 34 African countries with the highest vaccination coverage in 2022, a development the WHO in a May 2023 statement, described as a “dramatic achievement’.^5^

Consequently, the Tanzanian story provides a rare puzzle to unravel scientific evidence of the criticality of social determinants of health behaviour. While extensive research has illuminated the epidemiology of COVID-19 and the effectiveness of vaccines, less is known about the socio-ecological factors influencing vaccine uptake in Tanzania, particularly in the context of initial governmental scepticism towards vaccines. Therefore, it was imperative to investigate and establish whether socio-ecological factors, including government policies and regulations, institutions, communal influence, and interpersonal and intrapersonal factors, influenced the uptake of COVID-19 vaccination in Tanzania. This compendium is a collection of eight studies that aimed to underscore the socio-ecological determinants of COVID-19 vaccine uptake, using a cross-sectional approach. Collectively, the quantitative and qualitative empirical studies published here contribute to the understanding of how government policies and regulations, institutions, family ties, social relations, and personal knowledge provide a shared understanding of the factors that influenced the acceptance or rejection of COVID-19 vaccination during the pandemic.

Using quantitative and qualitative data from eight regions of Tanzania, the study explored how the socio-ecological model provides a comprehensive approach to understanding health behaviours, including vaccine uptake. This model posits that health behaviours are influenced by a complex interplay of factors at multiple levels: individual, interpersonal, community, institutional, and policy.^6^ Several studies have successfully applied this model to understand the determinants of vaccine uptake, revealing how each level contributes to overall vaccine acceptance and coverage.^7^

Four of the studies in this issue (Exploring determinants of COVID-19 vaccine uptake in Tanzania: A socio-ecological perspective; The role of social influence on COVID-19 vaccination hesitancy and acceptance in Tanzania; Sociocultural practices and COVID-19 prevention: A qualitative study of Mtwara, Shinyanga, and Arusha, Tanzania; ‘In God We Trust’: The role of religion in COVID-19 vaccinations in Dar es Salaam, Tanzania)^8,9,10,11^ underscore the importance of social cohesion even in matters of health. These studies examined the role played by social ambience, including social relationships, religion, and culture, in determining COVID-19 vaccination hesitancy and acceptance in Tanzania. Social cohesion is often described as the glue that holds society together, as an ‘attribute of a collective, indicating the quality of collective togetherness’.^12^

To gain a more nuanced understanding of the intrapersonal context of decision-making for health behaviours, two studies focused on perceptions of the risks of disease versus benefits of vaccinations as predictors of acceptance or hesitancy for COVID-19 vaccinations and bridging science and spirituality. The studies found that perceptions of the severity of the risks or benefits of vaccination and individual modes of spirituality significantly impacted the uptake of COVID-19 vaccination. The studies found that decision-making is influenced not only by empirical data but also by individuals’ health beliefs, norms, perceptions of self-efficacy, personal experiences, and social pressure for approval or belongingness. The findings of these studies are consistent with other studies (Benham, Atabati, Oxoby et al. and Wang, Chukwu, Mwanyika-Sando et al.) in which most survey respondents and interview participants who intended to receive a COVID-19 agreed that COVID-19 disease was severe, and vaccination was necessary^13,14^ as well as the study by Wang et al. on the acceptability of other vaccines linked acceptability to the perceived effectiveness and importance of vaccination.^15^

There was a critical unmet need for comprehensive research into how socio-ecological factors, such as personal beliefs and governmental policies, have influenced the dramatic change in vaccine uptake in Tanzania. For example, the national leadership of Tanzania announced on national mass media that the country ‘has no plans in place to accept COVID-19 vaccines’ and encouraged the populace to opt for alternative traditional dietary remedies and steam inhalation [*kujifukiza*] against globally prescribed vaccines.^16^ Hence, one of the articles in this volume has examined the lessons learned from the presidential endorsement of traditional medicine as an alternative to the COVID-19 vaccine. Although the practice of traditional medicine is not new in Africa, the campaign as an alternative remedy for COVID-19 treatment by the leadership of Tanzania was not only unprecedented but also played a role in determining the intention to vaccinate.

Furthermore, the roles of mass media and community engagement have been examined. The initial scepticism among Tanzania’s healthcare professionals and media as highlighted by Konje et al.^17^, underscores a significant gap in understanding role played by media on vaccine acceptance. Accordingly, the impact of media on behaviour has been well-documented corroborating other previous studies. For example, Saei, Valadi, Karimi, and Khammarnia opine that mass media, especially radio and television, are some of the main pillars for promoting public health education and information^18,19,20^ in society.

## Conclusion

In summary, the studies in this collection speak to all layers of the socio-ecological model of health behaviour, including intrapersonal, interpersonal, community, institution, and policy. In doing so, the authors collectively have contributed to building a foundation of innovative scientific knowledge on the socio-ecological determinants of health behaviour using COVID-19 vaccination in Tanzania as a reference. The authors warn that the medical monologue approach to public health campaigns, in which the provision of medicines and supplies are regarded as the most critical element for success, will continue to fail.
